# Supplementary figures and images for: Genomic Surveillance of Yellow Fever Virus Epizootic in São Paulo, Brazil, 2016 – 2018
Source: PLoS Pathog. 2020 Aug 7;16(8):e1008699. doi: 10.1371/journal.ppat.1008699 (PMC7437926; doi:10.1371/journal.ppat.1008699)

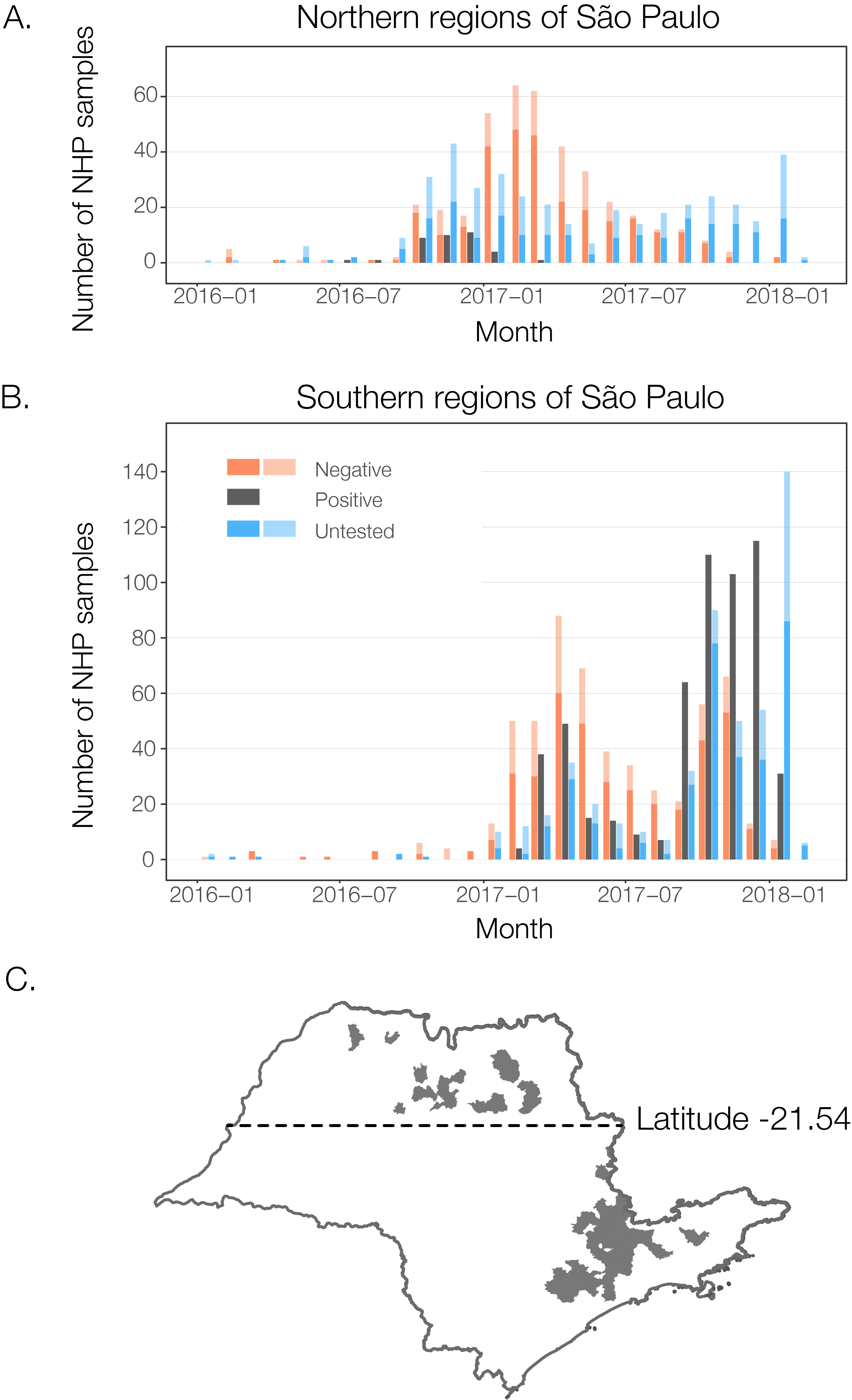

Supplement: S1 Fig — Reporting and testing of NHPs over time in northern (panel A) and southern (panel B) regions of São Paulo state. Here, municipalities where centroids fall north of latitude -21.54 are considered ‘northern’, and others are considered ‘southern’ (latitude indicated on panel C, along with locations of municipalities with positive cases in grey). This latitude was chosen to discriminate between locations most affected during phase 1, and those most affected during phase 2 and 3, and is therefore not central in São Paulo. Colours indicate results of testing. Darker shades of each colour in each bar represent results in testing in any municipality that detected positive NHPs during any month, and paler shades represent cases from those municipalities that never detected positive cases during the displayed period. Shapefiles used to produce this map are freely available from the Brazilian Institute of Geography and Statistics (IBGE) [42]. (TIF) [file ppat.1008699.s001.tif]

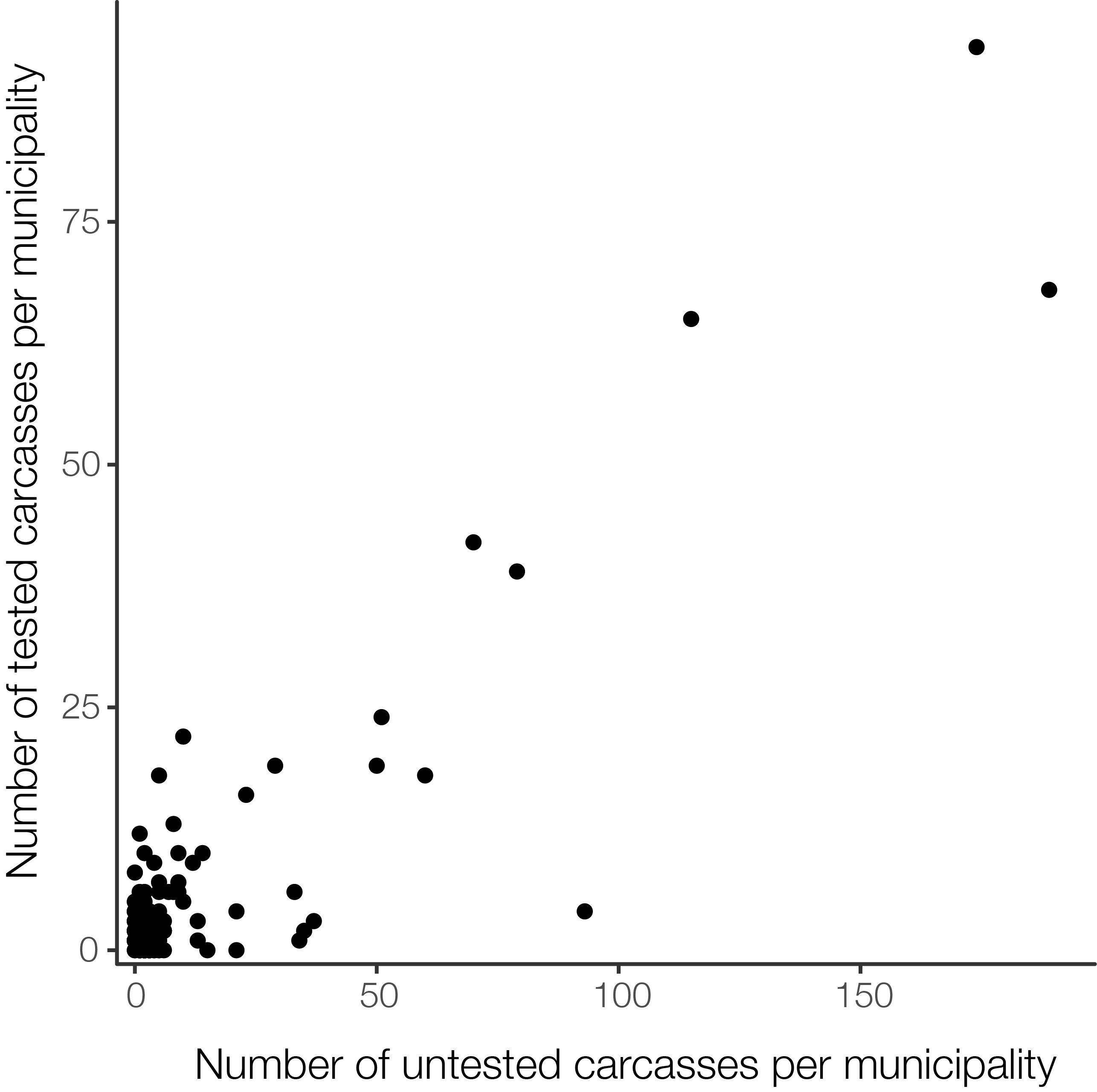

Supplement: S2 Fig — (TIF) [file ppat.1008699.s002.tif]

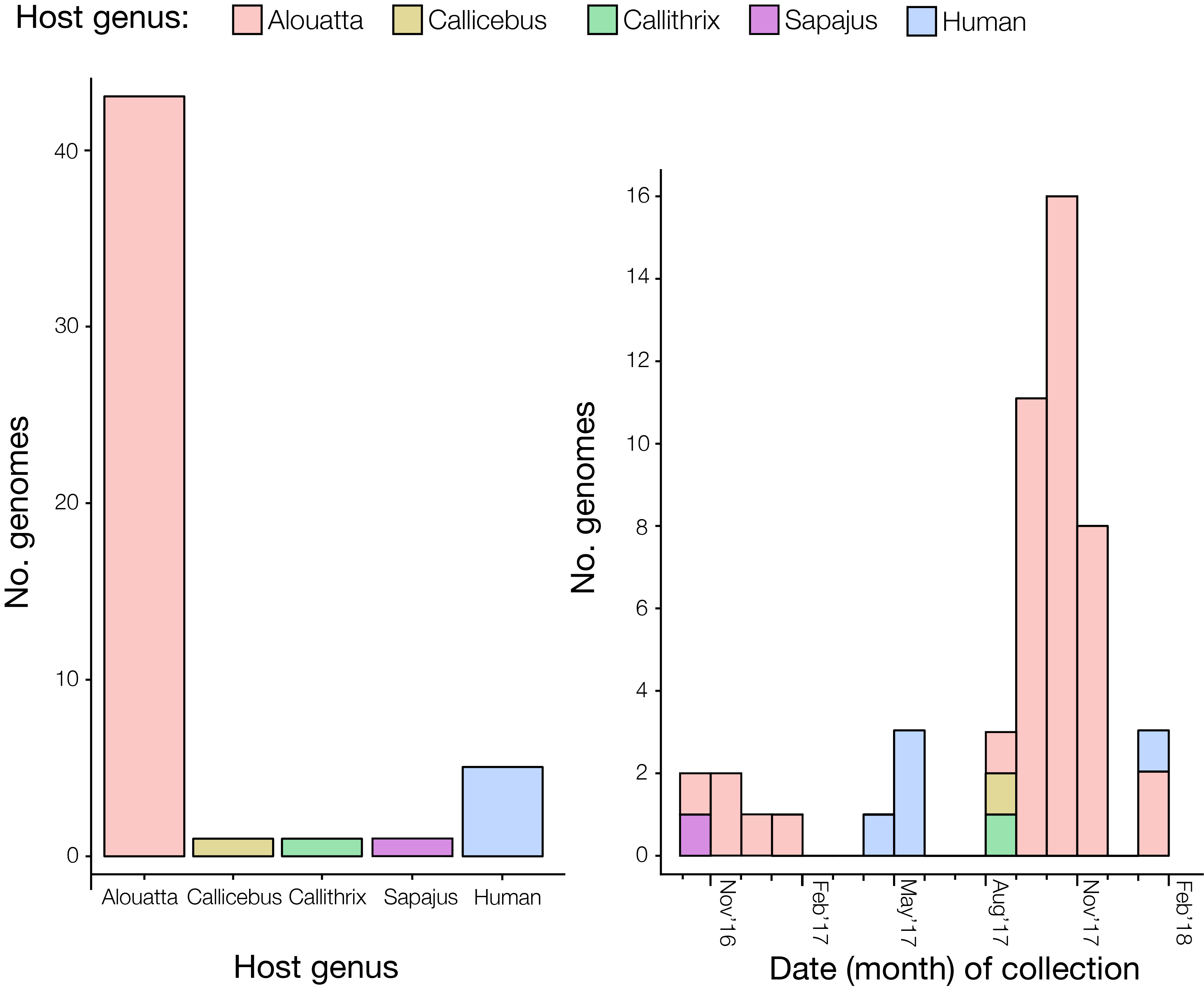

Supplement: S3 Fig — (TIF) [file ppat.1008699.s003.tif]

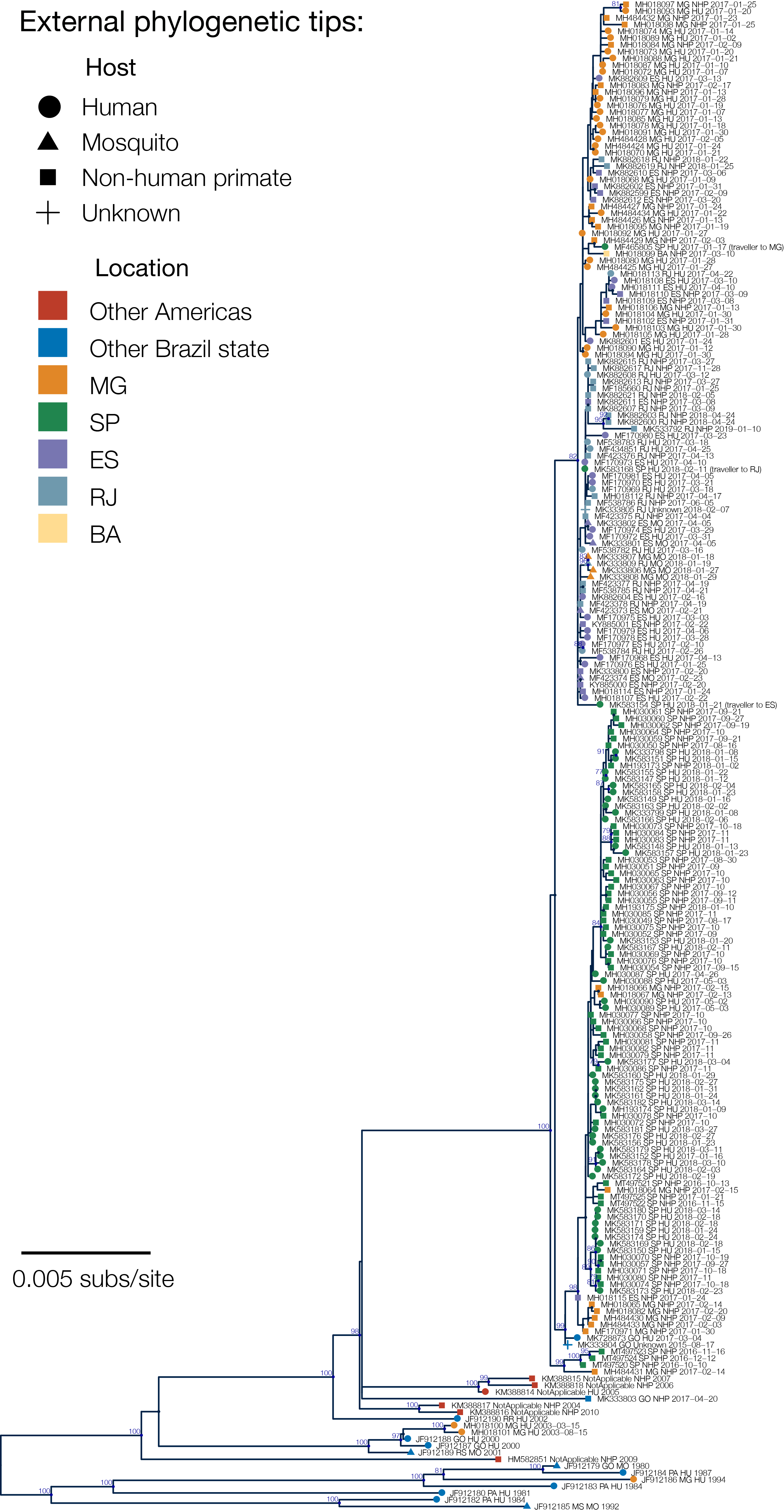

Supplement: S4 Fig — (TIF) [file ppat.1008699.s004.tif]
