# Supplementary material for: Genomic Surveillance of Yellow Fever Virus Epizootic in São Paulo, Brazil, 2016 – 2018
Source: PLoS Pathog. 2020 Aug 7;16(8):e1008699. doi: 10.1371/journal.ppat.1008699 (PMC7437926; doi:10.1371/journal.ppat.1008699)
Supplement: S1 Table — All sequences are from São Paulo. (DOCX) [file ppat.1008699.s005.docx]

**S1 Table.** Details of the YFV genome generated in this study. All sequences are from São Paulo.

| **Accession number** | **Sequence ID** | **Municipality** | **Host** | **Collection date** | **CT** |
| --- | --- | --- | --- | --- | --- |
| MT497520 | CP224 | Potirendaba | Sapajus | 10 Oct 2016 | 22 |
| MT497521 | CP159 | Pindorama | Alouatta | 13 Oct 2016 | 18 |
| MT497522 | CP182 | Jaboticabal | Alouatta | 15 Nov 2016 | 15 |
| MT497523 | CP227 | Catanduva | Alouatta | 16 Nov 2016 | 12 |
| MT497524 | CP164 | Catigua | Alouatta | 12 Dec 2016 | 14 |
| MT497525 | CP179 | Ribeirao Preto | Alouatta | 21 Jan 2017 | 16 |
| MH030087 | Y39 | Americana | Human | 26 April 2017 | 32 |
| MH030090 | Y42 | Sao Joao Boa Vista | Human | 02 May 2017 | 35 |
| MH030088 | Y40 | Tuiuti | Human | 03 May 2017 | 37 |
| MH030089 | Y41 | Sao Joao Boa Vista | Human | 03 May 2017 | 33 |
| MH030050 | Y2 | Louveira | Callicebus | 16 August 2017 | 11 |
| MH030049 | Y1 | Vinhedo | Callithrix | 17 August 2017 | 12 |
| MH030053 | Y5 | Itatiba | Alouatta | 30 August 2017 | 10 |
| MH030051 | Y3 | Louveira | Alouatta | Sept 17 | 9 |
| MH030052 | Y4 | Louveira | Alouatta | Sept 17 | 15 |
| MH030055 | Y7 | Jundiai | Alouatta | 11 Sept 2017 | 11 |
| MH030056 | Y8 | Jundiai | Alouatta | 12 Sept 2017 | 9 |
| MH030054 | Y6 | Jundiai | Alouatta | 15 Sept 2017 | 12 |
| MH030062 | Y14 | Itatiba | Alouatta | 19 Sept 2017 | 12 |
| MH030059 | Y11 | Itatiba | Alouatta | 21 Sept 2017 | 12 |
| MH030061 | Y13 | Itatiba | Alouatta | 21 Sept 2017 | 13 |
| MH030058 | Y10 | Itatiba | Alouatta | 26 Sept 2017 | 10 |
| MH030057 | Y9 | Braganca Paulista | Alouatta | 27 Sept 2017 | 16 |
| MH030060 | Y12 | Itatiba | Alouatta | 27 Sept 2017 | 12 |
| MH030063 | Y15 | Itatiba | Alouatta | Oct 17 | 11 |
| MH030064 | Y16 | Jundiai | Alouatta | Oct 17 | 10 |
| MH030065 | Y17 | Jundiai | Alouatta | Oct 17 | 12 |
| MH030066 | Y18 | Jundiai | Alouatta | Oct 17 | 11 |
| MH030067 | Y19 | Jundiai | Alouatta | Oct 17 | 24 |
| MH030068 | Y20 | Jundiai | Alouatta | Oct 17 | 12 |
| MH030069 | Y21 | SaoPaulo | Alouatta | Oct 17 | 16 |
| MH030072 | Y24 | Jarinu | Alouatta | Oct 17 | 10 |
| MH030075 | Y27 | Jarinu | Alouatta | Oct 17 | 19 |
| MH030076 | Y28 | Jarinu | Alouatta | Oct 17 | 17 |
| MH030077 | Y29 | Jarinu | Alouatta | Oct 17 | 16 |
| MH030078 | Y30 | Morungaba | Alouatta | Oct 17 | 12 |
| MH030071 | Y23 | Campo Limpo Paulista | Alouatta | 18 Oct 2017 | 21 |
| MH030073 | Y25 | Mairipora | Alouatta | 18 Oct 2017 | 24 |
| MH030074 | Y26 | Jarinu | Alouatta | 18 Oct 2017 | 15 |
| MH030070 | Y22 | Campo Limpo Paulista | Alouatta | 19 Oct 2017 | 14 |
| MH030079 | Y31 | Nazare Paulista | Alouatta | Nov 17 | 15 |
| MH030080 | Y32 | Nazare Paulista | Alouatta | Nov 17 | 14 |
| MH030081 | Y33 | Campo Limpo | Alouatta | Nov 17 | 25 |
| MH030082 | Y34 | Campo Limpo | Alouatta | Nov 17 | 16 |
| MH030083 | Y35 | Campo Limpo | Alouatta | Nov 17 | 15 |
| MH030084 | Y36 | Piracaia | Alouatta | Nov 17 | 13 |
| MH030085 | Y37 | Piracaia | Alouatta | Nov 17 | 14 |
| MH030086 | Y38 | Piracaia | Alouatta | Nov 17 | 15 |
| MH193173 | SA129 | Guarulhos | Alouatta | 02 Jan 2018 |  |
| MH193174 | SA130 | Mairipora | Human | 09 Jan 2018 |  |
| MH193175 | SA131 | Sao Paulo | Alouatta | 10 Jan 2018 |  |
