# Supplementary material for: Genomic Surveillance of Yellow Fever Virus Epizootic in São Paulo, Brazil, 2016 – 2018
Source: PLoS Pathog. 2020 Aug 7;16(8):e1008699. doi: 10.1371/journal.ppat.1008699 (PMC7437926; doi:10.1371/journal.ppat.1008699)
Supplement: S2 Table — Detailed information for each sequenced isolate can be found in S1 Table. (DOCX) [file ppat.1008699.s006.docx]

**S2 Table.** Non-human primate yellow fever virus genome sequences from São Paulo, by host genus. Detailed information for each sequenced isolate can be found in **S1 Table**.

| **Host genus** | **Confirmed NHP cases*** | | | **Genomes generated** | | | **RT-qPCR**  **Ct values** | | | **Sequence coverage** | | |
| --- | --- | --- | --- | --- | --- | --- | --- | --- | --- | --- | --- | --- |
|  | Total | % of total** | | Total | % of total | | Median | | Range | Median | | Range |
| Alouatta | 403 | | 88 | 43 | | 84 | 14 | 9-25 | | 99.3 | 86.1-99.4 | |
| Callicebus | 9 | | 2 | 1 | | 2 | 11 | n.a. | | 99.3 | n.a. | |
| Callithrix | 35 | | 8 | 1 | | 2 | 12 | n.a. | | 99.2 | n.a. | |
| Cebidae | 9 | | 22 | 0 | | 0 | n.a. | n.a. | | n.a. | n.a. | |
| Sapajus | 3 | | 0.7 | 1 | | 2 | 22 | n.a. | | 96.3 | n.a. | |
| Human | - | | - | 5 | | 10 | 34 | 32-37 | | 96.1 | 80.8-99.3 | |

*Cases reported to Instituto Adolfo Lutz, São Paulo, from week 29 of 2016 through week 4 of 2018. n.a.=not applicable.

**For which genera was known
