# Supplementary material for: Genomic Surveillance of Yellow Fever Virus Epizootic in São Paulo, Brazil, 2016 – 2018
Source: PLoS Pathog. 2020 Aug 7;16(8):e1008699. doi: 10.1371/journal.ppat.1008699 (PMC7437926; doi:10.1371/journal.ppat.1008699)
Supplement: S3 Table — (DOCX) [file ppat.1008699.s007.docx]

**S3 Table:** Mean branch dispersal velocity estimates.

| **Dataset (number of sequences)** | **Mean branch dispersal velocity (km/day, [95% HPD]) based on phylogenetic branches in defined phases** | | | |
| --- | --- | --- | --- | --- |
|  | **All** | **Phase 1: root up to 2017-02-01** | **Phase 2: 2017-02-01 to 2017-07-01** | **Phase 3: 2017-07-01 onwards** |
| **Full (99)** | 0.95 (0.53-2.42) | 1.87 (0.64-5.95) | 1.05 (0.23-2.89) | 0.80 (0.44-1.85) |
| **Geographically restricted (95)** | 0.83 (0.50-1.53) | 0.86 (0.40-1.94) | 0.93 (0.27-2.58) | 0.87 (0.46-1.79) |
